# MicroRNAs Associated with the Efficacy of Photodynamic Therapy in Biliary Tract Cancer Cell Lines

**DOI:** 10.3390/ijms151120134

**Published:** 2014-11-05

**Authors:** Andrej Wagner, Christian Mayr, Doris Bach, Romana Illig, Kristjan Plaetzer, Frieder Berr, Martin Pichler, Daniel Neureiter, Tobias Kiesslich

**Affiliations:** 1 Department of Internal Medicine I, Paracelsus Medical University/Salzburger Landeskliniken (SALK), Muellner Hauptstrasse 48, Salzburg 5020, Austria; E-Mails: ch.mayr@salk.at (C.M.); doris.bach@gmx.at (D.B.); f.berr@salk.at (F.B.); t.kiesslich@salk.at (T.K.); 2 Institute of Pathology, Paracelsus Medical University/Salzburger Landeskliniken (SALK), Salzburg 5020, Austria; E-Mails: r.illig@salk.at (R.I.); d.neureiter@salk.at (D.N.); 3 Laboratory of Photodynamic Inactivation of Microorganisms, Department of Materials Science and Physics, University of Salzburg, Salzburg 5020, Austria; E-Mail: kristjan.plaetzer@sbg.ac.at (K.P.); 4 Division of Oncology, Medical University Graz, Graz 8036, Austria; E-Mail: martin.pichler@medunigraz.at (M.P.); 5 Department of Experimental Therapeutics, the University of Texas MD Anderson Cancer Center, Houston, TX 77054, USA; 6 Institute of Physiology and Pathophysiology, Paracelsus Medical University, Salzburg 5020, Austria

**Keywords:** MicroRNAs, bile duct cancer, photodynamic therapy, cytotoxicity, sensitivity

## Abstract

Photodynamic therapy (PDT) is a palliative treatment option for unresectable hilar biliary tract cancer (BTC) showing a considerable benefit for survival and quality of life with few side effects. Currently, factors determining the cellular response of BTC cells towards PDT are unknown. Due to their multifaceted nature, microRNAs (miRs) are a promising analyte to investigate the cellular mechanisms following PDT. For two photosensitizers, Photofrin^®^ and Foscan^®^, the phototoxicity was investigated in eight BTC cell lines. Each cell line (untreated) was profiled for expression of *n* = 754 miRs using TaqMan^®^ Array Human MicroRNA Cards. Statistical analysis and bioinformatic tools were used to identify miRs associated with PDT efficiency and their putative targets, respectively. Twenty miRs correlated significantly with either high or low PDT efficiency. PDT was particularly effective in cells with high levels of clustered miRs 25-93*-106b and (in case of miR-106b) a phenotype characterized by high expression of the mesenchymal marker vimentin and high proliferation (cyclinD1 and Ki67 expression). Insensitivity towards PDT was associated with high miR-200 family expression and (for miR-cluster 200a/b-429) expression of differentiation markers Ck19 and Ck8/18. Predicted and validated downstream targets indicate plausible involvement of miRs 20a*, 25, 93*, 130a, 141, 200a, 200c and 203 in response mechanisms to PDT, suggesting that targeting these miRs could improve susceptibility to PDT in insensitive cell lines. Taken together, the miRNome pattern may provide a novel tool for predicting the efficiency of PDT and—following appropriate functional verification—may subsequently allow for optimization of the PDT protocol.

## 1. Introduction

Prognosis of patients with biliary tract cancer (BTC) is generally poor due to limited therapeutic options [1,2]. For hilar BTC, resection (R0) is the only curative treatment—however, only 20%–30% of patients are eligible for surgery owing to local tumor extension [3]. Photodynamic therapy (PDT) has been used in prospective clinical studies for palliative treatment of unresectable hilar BTC resulting in a median survival time between 12 and 21 months and better quality of life [4,5]. In a retrospective study including 184 patients, palliative PDT was comparable with incomplete R1/R2 resection, but was associated with reduced rate of complications and peri-procedural side effects for the patient [6]. As summarized by Gao *et al.* [7], PDT offers a considerable benefit for survival and quality of life with few side effects and thus should be offered to patients with unresectable hilar BTC. In this context, two main photosensitizers (PSs) have been successfully established for routine use in the clinic, *i.e.*, hematoporphyrin derivative (porfimer sodium/Photofrin^®^) and meso-tetrahydroxyphenylchlorine (mTHPC/Foscan^®^) [7,8].

PDT relies on the combination of two individually non-toxic agents, visible light and a PS, which after the PS has been taken up by the tumor cells and is subsequently activated by light, results in the production of reactive oxygen species (ROS) and cell death [9,10]. The severity of damage, *i.e.*, the PDT dose is most important in determining the mode of cellular response (survival or induction of apoptosis *versus* necrosis [11]). The acute oxidative stress response and cell damage induced by PDT are not dependent on the cellular signaling factors that may cause resistance in the context of chemotherapy [8].

In our previous studies, we demonstrated the phototoxicity of PDT in different human BTC cell lines to be highly heterogeneous [12,13] and preferentially linked to low differentiation and proliferation of the cells. Although phototoxic effects were significantly correlated to the uptake of PS, a discrepancy between PS uptake and final cytotoxicity could be observed with no actual explanation existing yet [13]. These results indicate that additional molecular factors determine the overall cytotoxic efficiency of PDT in BTC cells.

MicroRNAs (miRs) form a class of regulatory non-coding RNAs that are widely expressed in all metazoan eukaryotes. They are about 22 nucleotides long and bind their target mRNAs via partial base pairing resulting in translational silencing [14]. Human cancer is associated with changes in miR expression [15,16]. It was reported that the pattern of miR expression varies dramatically across tumor types and that miR-profiles reflect the developmental lineage and differentiation state of a tumor [17]. Of special importance, this class of molecules is now intensively being studied in regard to their association with chemo- and radiosensitivity with the aim to predict or modulate the individual tumor’s responsiveness towards these treatments based on its miRNome, *i.e.*, the comprehensive miR expression pattern (recently reviewed in [18,19,20,21,22,23]).

Currently, the factors determining the cellular response of BTC cells towards PDT are unknown. Since the mode of operation of PDT is fundamentally different from chemo- or radiotherapy, an inherent or acquired resistance to the latter does not affect the sensitivity towards PDT [9]. Due to the multifaceted regulatory roles of miRs, the miRNome yet has the potential to explain the observed heterogeneity in cytotoxic response and may provide a novel tool for predicting the efficiency of PDT in each particular case and probably to adjust the individual treatment protocol.

The hypothesis in this project is that specific differences in the miRNome between individual BTC cell lines account for the observed differences in susceptibility towards PDT. This hypothesis is tested in eight different BTC cell lines by comprehensive analysis of the miR expression profile and its relationship towards PDT efficiency and expression of markers of differentiation or proliferation. The results may provide pilot data on the potential of miRs as modifiers of the therapeutic efficiency of PDT.

## 2. Results and Discussion

### 2.1. Results

#### 2.1.1. Phototoxicity Experiments

**Figure 1:**
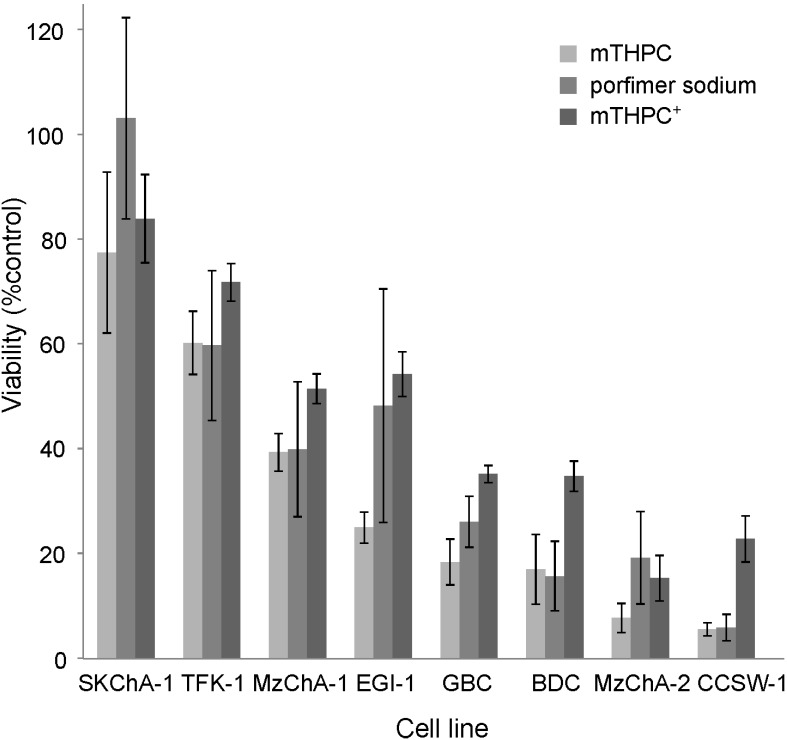
Viability of BTC (biliary tract cancer) cell lines following PDT (photodynamic therapy) with mTHPC or porfimer sodium. PDT treatment was performed after 20 h incubation with 0.588 µM (400 ng·mL^−1^) mTHPC or 1.2 µg·mL^−1^ (2 µM) porfimer sodium by illumination with 0.65 J/cm^2^ (660 nm, mTHPC) and 4.30 J/cm^2^ (624 nm, porfimer sodium). ^+^ raw data as previously published [13], reproduced by permission of The Royal Society of Chemistry (RSC) on behalf of the European Society for Photobiology, the European Photochemistry Association, and RSC.

Twenty-four hours after illumination with an intermediate energy dose of 650 mJ/cm^2^ at 660 nm for mTHPC and 4300 mJ/cm^2^ at 624 nm for porfimer sodium, respectively, the cell lines displayed large variation in the degree of toxicity (Figure 1) with comparable results for the two photosensitizers (PSs). Extreme differences between cell lines were obtained for CCSW-1, of which 94% (surviving fraction 6%) and SkChA-1, of which 0%–22% (surviving fraction ≥ 78%) are killed by photodynamic treatment, respectively. A grouping of cell lines with different susceptibility towards photodynamic treatment could be observed: cell lines EGI-1, GBC, MzChA-1, SkChA-1, and TFK-1 showed relatively low phototoxic effects, whereas CCSW-1, BDC, and MzChA-2 showed considerable higher phototoxic efficiency (Figure 1). Accordingly, correlation analysis of the phototoxicity values obtained for the two PSs revealed a highly significant correlation (*p* < 0.01; Pearson two-tailed correlation, data not shown).

#### 2.1.2. MicroRNA Expression Profiling

Analysis of 754 miRs using unique Taqman assays resulted in miR expression patterns for each cell line, wherein 332 (BDC cells) up to 445 (MzChA-1 cells) individual miRs could be detected. Expression of 170 miRs was below the quantification threshold in the eight BTC cell lines (Table 1). Supplemental data file 1 lists detailed expression data of 395 miRs, which were quantifiable in at least four cell lines.

**Table 1:** MiR-profiling in 8 BTC cell lines.

Cell Line	*n* miRs	*n* Cell Lines	*n* miRs
BDC	332	8	200
CCSW	379	7	62
EGI	352	6	48
GBC	349	5	37
MzChA1	416	4	48
MzChA2	445	3	37
SkChA1	412	2	56
TFK	359	1	96
-		0	170
		total	754

#### 2.1.3. PDT Efficiency and MicroRNA Expression

**Figure 2:**
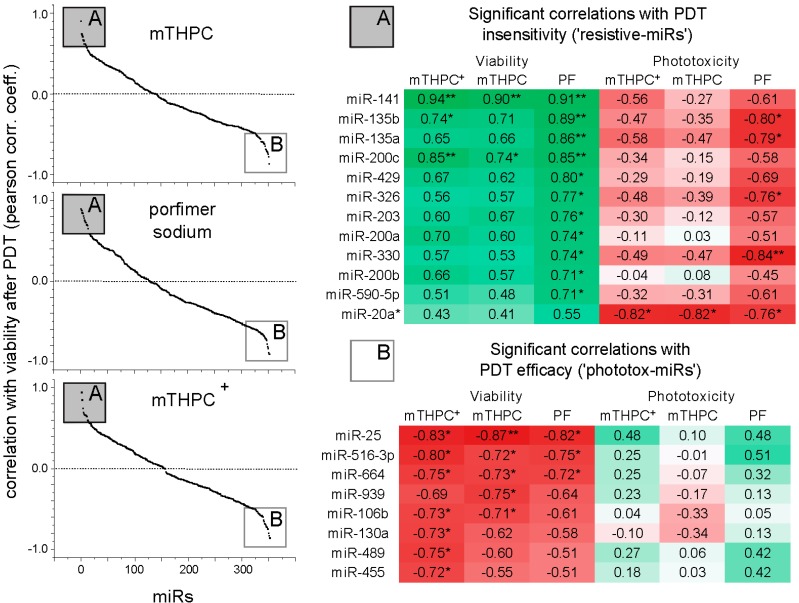
Correlations of miR expression, viability and uptake-corrected phototoxicity after PDT with mTHPC or porfimer sodium. Pearsons correlation coefficient of 395 miRs expressed in at least four cell lines. 20 miRs correlate significantly either with PDT insensitivity (A, positive correlation with viability after PDT) or phototoxicity (B, negative correlation with viability after PDT). The color indicates positive (green) and negative (red) correlation coefficients. Correlations with viability and (uptake-corrected) phototoxicity show reciprocal trends. Of note, miR-20a* correlates significantly only with the three data sets for uptake-corrected phototoxicity. ^+^ Based on phototoxicity raw data as previously published [13], reproduced by permission of The Royal Society of Chemistry (RSC) on behalf of the European Society for Photobiology, the European Photochemistry Association, and RSC; the full list of correlations is provided in supplementary file 2. * *p* < 0.05; ** *p* < 0.01; abbreviations: PF: porfimer sodium.

The correlation of miR expression levels and viability after PDT using the three data sets for the two PSs in the BTC cell lines is shown in Figure 2. To correct for cell line-specific differences in PS uptake, correlation analysis between (uncorrected) viability data sets are supplemented by the corresponding photoxicity data corrected for the relative uptake of the respective PS. Only those miRs were included in the correlation analysis, which were quantifiable in at least four cell lines (*n* = 395).

**Figure 3:**
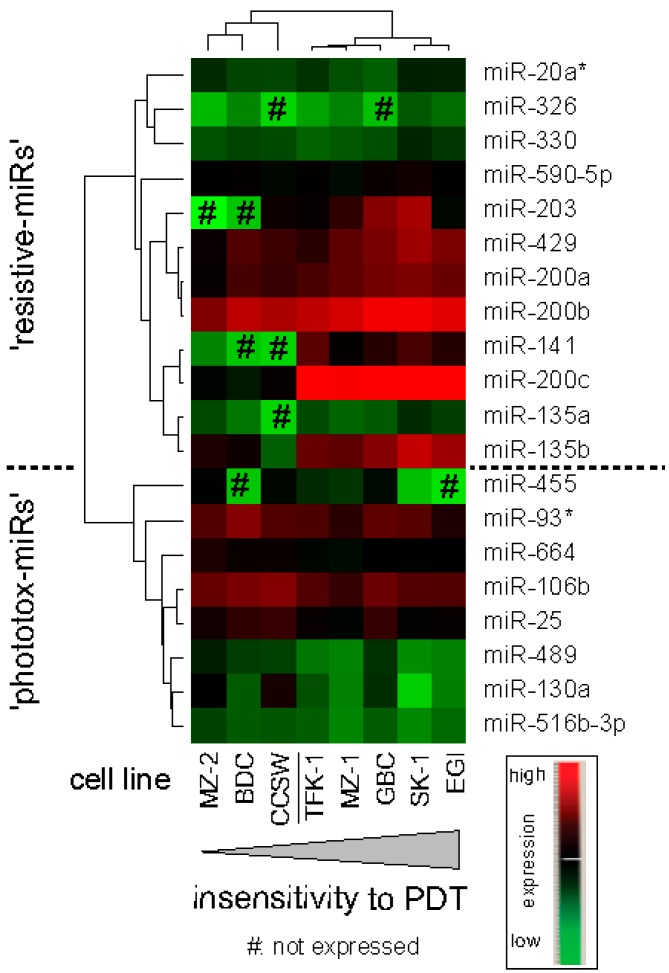
Expression of miRs significantly correlating with PDT. Hierarchical clustering reveals two clusters of cell lines: five differentiated/PDT-resistive and three less differentiated/PDT-sensitive cell lines. Some miRs (141, 203, 326, 455 and 135a) are not expressed in PDT-sensitive (clustered) cell lines (CCSW, MZ-2 and BDC).

As shown in Figure 2, twelve miRs showed significant positive correlation with the viability (in case of miR-20a*, only with all three data sets of uptake-corrected phototoxicity) after PDT treatment and eight miRs were negatively correlated with the viability signal, *i.e.*, their expression was associated with higher cytotoxicity (phototoxicity-associated miRs). These groups of miRs are subsequently referred to as “resistive-miRs” and “phototox-miRs”, respectively. As indicated in Figure 2, the correlation analysis results based on uptake-corrected phototoxicity show similar trends (yet not significant). Some miRs showed significant association consistent for all three PDT viability data sets; in the other cases, the correlation was not significant for one or two of the other data sets but showed a similar trend. Among the 12 “resistive-miRs”, all five members of miR-200 family (highly significant: miR-141 and miR-200c) and the two miR-135 family members showed significant correlation with PDT insensitivity. The highest level of significance among “phototox-miRs” was obtained for clustered miRs 25-93*-106b, miRs 516-3p and 664. A heatmap and hierarchical clustering of miR expression profiles based on significant results is shown in Figure 3. MiR expression *vs.* viability is plotted in supplemental data file 3 (Figure S1) for all significant correlations confirming a direct or indirect proportional association. A list of all calculated correlations is provided in supplemental data file 2.

#### 2.1.4. Correlation Analysis: miR Expression and PDT Efficiency, PS Uptake, Glutathione (GSH) Levels and Markers of Differentiation and Proliferation

In two previous studies by our group [13,24] the cell lines were comprehensively analyzed for mTHPC uptake, GSH levels and immunochemical characteristics. Correlation of these parameters with miR expression measured in the current study yielded the following significant correlations (Table 2): “Resistive-miRs” (except for miR-20a*) correlate negatively with PS uptake, vimentin and markers of proliferation (CyclinD1 and Ki67; except for miR-590-5p). For all “resistive-miRs” (except for miR-20a*), Table 2 indicates a (partly significant) positive correlation with markers of differentiation (Ck-19 and Ck-8/18). “Phototox-miRs” correlate positively and in part significantly with (i) PS uptake; (ii) expression of CyclinD1 and Ki67 (except for miRs 25, 130a and 455); and (iii) vimentin in case of miRs 516-3p, 664 and 106b. For markers of differentiation (Ck-19 and Ck-8/18), a (not significant) negative correlation can be observed for “phototox-miRs” 130a, 516-3p and 455. MiR-130a correlates inversely with GSH (*p* < 0.05) and Ck-19 (*p* = 0.054).

#### 2.1.5. *In Silico* Target Prediction and Bioinformatic Approach

**Figure 4:**
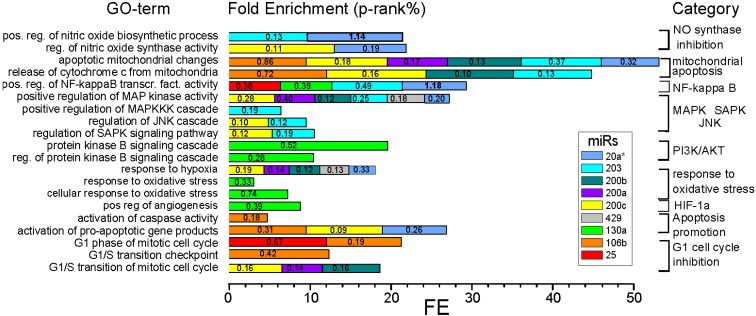
Comparison of PDT-relevant GO terms with enriched miR targets following *in silico* prediction. Predicted and validated targets of eight significant miRs are enriched in PDT relevant GO terms using the DAVID functional chart annotation tool (*p*-rank%: 10/(ranking of *p*-value of GO term × (100/n predicted and validates targets)).

*In silico* target prediction revealed 6865 mRNA targets for the 20 miRs significantly correlating with viability after PDT. The Database for Annotation, Visualization and Integrated Discovery (DAVID) functional chart annotation revealed a total of 3084 Gene-Ontology (GO) terms (supplemental data files 4 and 5). Nine miRs (“resistive-miRs” 20a*, 200a, 200b, 200c, 203, 429 and “phototox-miRs” 25, 106b and 130a) showed involvement in PDT-relevant GO terms, e.g., concerning mitochondrial apoptosis, nitric oxide (NO) synthase regulation, regulation of NF-κB or stress activated protein kinase signaling. Comparison of Fold Enrichment and EASE score for all PDT-related GO terms for these nine miRs is depicted in Figure 4. Supplementary data file 4 contains a list of all predicted and validated targets. Finally, a detailed summary of combined target analysis following literature search and associated GO-terms is listed in supplemental data file 6 (Table S1).

As summarized in Table 3, an accumulation of PDT-related targets can be observed for “phototox-miR”-130a targeting PRDX3, SESN2, HIF-1α (together with miRs 141, 200c, 135a), AKT1 (together with miRs 106b and 93*), NOS3, SIRT7 and SIRT6; targets of this miR are enriched in GO terms “response to oxidative stress”, “protein kinase B signaling”, “positive regulation of NF-κB transcription factor activity” (together with miRs 25 and 203), and “positive regulation of angiogenesis”. Furthermore, no contradictive results, *i.e.*, targets, which would possibly decrease PDT efficiency if inhibited by this “phototox-miR”, could be found.

MiR-25 (which shows highest level of significance in correlation with PDT toxicity) targets AKT2, SESN3, BCL2, NOX4, HMOX2, and TRAF6. It showed target enrichment in “G1 phase of mitotic cell cycle” and “positive regulation of NF-κB transcription factor activity”. Mir-93* targets AKT1, SESN2, NOX4, NFE2 and MGST1, which could possibly contribute to a pro-apoptotic function in the context of PDT.

**Table 2:** Correlation analysis ^a,b^—miR expression and markers of proliferation and differentiation, GSH levels and PS uptake ^c^.

(Phenotypic) Markers	Resistive-miRs												Phototox-miRs							
	141	135b	135a	200c	429	326	203	200a	330	200b	590-5p	20a*	25	516b-3p	664	93*	106b	130a	489	455
**Uptake**	−0.70	−0.60	−0.47	**−0.74 ^†^**	−0.63	−0.43	−0.55	**−0.74 ^†^**	−0.39	**−0.74 ^†^**	−0.52	−0.03	0.65	**0.72 ^†^**	0.67	0.64	**0.79 ^†^**	**0.94 ^††^**	0.54	0.65
**Ck19**	0.24	0.50	−0.01	0.23	**0.72 ^†^**	0.11	0.50	0.66	0.14	**0.75 ^†^**	0.17	−0.19	−0.30	−0.63	−0.33	−0.05	−0.36	−0.70	−0.29	−0.54
**Ck8/18**	0.39	0.64	0.37	0.35	**0.72 ^†^**	0.39	0.11	0.59	0.48	**0.77 ^†^**	**0.73 ^†^**	0.35	−0.18	−0.40	−0.04	0.09	−0.09	−0.55	−0.03	−0.68
**E-Cadherin**	0.51	0.52	0.27	0.47	0.40	0.06	−0.05	0.49	−0.10	0.53	0.34	0.25	−0.16	−0.39	−0.21	−0.19	−0.37	−0.34	−0.16	−0.64
**Vimentin**	−0.58	−0.66	−0.39	−0.64	−0.44	−0.18	−0.38	−0.67	0.24	−0.51	−0.07	0.05	0.47	0.67	**0.78 ^†^**	0.53	**0.78 ^†^**	0.45	0.42	0.18
**Cyclin D1**	−0.42	−0.42	−0.17	−0.23	−0.09	0.08	−0.45	−0.29	0.57	−0.21	0.00	0.35	0.40	0.38	0.63	0.64	0.70	0.26	0.11	−0.21
**Ki67**	−0.58	−0.38	−0.15	−0.53	−0.23	0.06	**−0.78 ^†^**	−0.48	0.40	−0.30	0.20	0.08	0.58	0.65	**0.78 ^†^**	0.65	**0.73 ^†^**	0.40	**0.73 ^†^**	−0.05
**GSH**	0.24	0.21	0.11	0.20	0.27	0.12	0.40	0.29	0.12	0.33	0.36	−0.19	−0.15	−0.18	−0.06	−0.21	−0.33	**−0.72 ^†^**	−0.16	−0.44

^a^ Pearson’s correlation coefficient of 395 miRs expressed in at least four cell lines, ^†^ *p* < 0.05, ^††^ *p* < 0.01; ^b^ negative correlations are marked with red color; ^c^ mTHPC uptake (fluorimetry) and other parameters as previously published [13], reproduced by permission of The Royal Society of Chemistry (RSC) on behalf of the European Society for Photobiology, the European Photochemistry Association, and RSC.

“Resistive-miR”-141 (which showed the best correlation with insensitivity to PDT) and miR-200a reveal BACH1, KEAP1and NOX1 as targets, whose inhibition by this “resistive-miRs” could possibly impair PDT efficacy. Interestingly, also HIF-1α, VEGFA, HSPA4L and LOX are targets of these miRs (with a theoretically controversial effect). MiR-141 has no enrichment in PDT-relevant GO term categories, miR-200a in “apoptotic mitochondrial changes”, “positive regulation of MAP kinase activity”, “response to hypoxia” and “G1/S transition of mitotic cell cycle”, which could correspond to a PDT-opposing function of this miR.

“Resistive-miR”-200c shows a contradictive accumulation of PDT-related targets as BAX, HIF-1α, VEGFA, BCL2, which would possibly improve PDT efficacy if inhibited by this “resistive-miR” (except for BAX). Similar discrepancies can be observed for “resistive-miRs” 20a*, 203, 141 and 200a. However, GO term-analysis shows clear involvement of miRs 20a*, 203 and 200c in PDT-relevant categories, as mitochondrial apoptosis, NO synthase regulation, regulation of NF-κB or stress activated protein kinase signaling (supplemental data file 6 (Table S1), Figure 4).

**Table 3:** Summary of combined bioinformatic approach and literature search after *in silico* target prediction for phototox- and resistive-miRs.

Function	MiR	Possible Impact on PDT Efficiency via:	Association with Cancer Phenotype
**“Phototox-miRs”**	**-130a**	neg. corr. with GSH (*), targets: PRDX3, SESN2, HIF-1α, SIRT6/7, NOS3, AKT1, GO: pos. reg. angiogenesis, protein B kinase signaling, pos. reg. NF-κB transcription factor activity	-
	**-25**	targets: AKT2, SESN3, BCL2, NOX4, HMOX2, GO: pos. reg. NF-κB transcription factor activity, G1/S cycle inhibition	induction of G1/S arrest via Wnt-inhibition (β-cat) [25] Associated with aggressive phenotype (e.g., gastric cancer) [26,27]
	-93*	targets: AKT1, SESN2, NOX4, NFE2, MGST1, Influence on Sp1 and Nrf2 TF [28,29] and PTEN/Akt signaling pathway [30]	Associated with aggressive phenotype (e.g., gastric and breast cancer) [26,27]
**“Resistive-miRs”**	**-141**	targets: BACH1, KEAP1, TGF-β1, Nrf2, NOX1	GO: EMT
	**-200a**	GO: apoptotic mitochondrial changes, pos. regulation of MAP kinase activity, targets: KEAP1, activation of Nrf2 and NAD(P)H-quinone oxidoreductase 1 [31]	suppression of Wnt (β-cat) and anti-proliferative function [32], miR family 200 is negatively associated with EMT (ZEB1/ZEB2) [33,34,35]
	**-200c/-203**	target: BAX, GO: apoptotic mitochondrial changes, release of cytochrome c from mitochondria, activation of proapoptotic gene products, pos. regulation of MAPKKK cascade, regulation of c-Jun *N*-terminal kinases (JNK) cascade, regulation of stress-activated protein kinase signaling pathway	neg. corr. with Ki67 (*), GO: EMT, 200c: targets CD44 [36,37], which is associated with high ROS levels and EMT [38], 203: “anti-stemness-miR”, which is down-regulated by CD44 [39,40]
	20a*	GO: activation of pro-apoptotic gene products, apoptotic mitochondrial changes, positive regulation of MAP kinase activity	-

Abbreviations: GO: enriched targets in GO terms; target: predicted or validated targets after *in silico* prediction; corr.: correlation with markers of proliferation and differentiation (Table 2); pos.: positive; neg.: negative; reg.: regulation; * *p* < 0.05, β-cat: beta-catenin; EMT: epithelial-mesenchymal-transition; ROS: reactive oxygen species; TF: transcription factors.

### 2.2. Discussion

In the actual study, several miRs showed either significant correlation with mTHPC- or porfimer-based phototoxicity (referred to as “phototox-miRs”, primarily miR cluster 25-93*-106b, miRs 130a, 516-3p and 664) or PDT insensitivity (referred to as “resistive-miRs”, mainly miR families 200, 135 and miRs 203, 330 and 326) in a set of eight BTC cells.

PDT is established for palliative treatment of BTC using mainly two photosensitizers (PSs): porfimer sodium (Photofrin^®^) and mTHPC (Foscan^®^). Although phototoxic effects were significantly correlated to the uptake of the PSs in our previous studies [13], a discrepancy between PS uptake and final cytotoxicity could be observed, indicating that additional molecular factors determine the overall cytotoxic efficiency of PDT in BTC cells. To further investigate the cellular mechanisms governing PDT efficiency, we compared the expression of miRs to the viability data obtained after *in vitro* PDT treatment using these two PSs in a model system of eight BTC cell lines. Based on the assumption that an overlapping set of miRs correlating with phototoxicity is more expressive if obtained after comparison with more than one set of phototoxicity data, we included the previously obtained data [13] in the statistical analysis. Of note, different passages of cells were compared and actual results were related to the initial viability values (at time of illumination) whereas data from our previous study [13] were related to *untreated* control cells 24 h post illumination. Nevertheless, only small differences in the absolute viability values were obtained for the three treatment conditions—confirming and reproducing the overall trend and the overall phototoxicity for each cell line. Accordingly, there was a clear overlap of significantly correlating miRs for the three treatment sets. If raw phototoxicity data were corrected for uptake and correlated with miR expression, these results could essentially be confirmed (Figure 2 and supplementary file 2). Therefore, we suggest the similar cell line-dependent differences seen for all three treatments are not caused by different modes of actions of these PSs, but are rather related to overall different cellular susceptibilities towards PDT probably caused by differential miR expression. So far, only two studies investigated the effects of PDT on particular miR expression levels after treatment in cell culture experiments [41,42]. None of the miRs significantly altered after PDT in the mentioned publications correlated clearly with PDT efficacy in the actual analysis. This might be explained by the different experimental settings (differences in PDT efficacy as a function of miR expression in different cell lines *vs.* changes of miR expression following PDT in a single cell line) and the different cell types used.

#### 2.2.1. MiR Expression and PDT Insensitivity

In the context of PDT, promising therapeutic strategies targeting cytoprotective mechanisms and increasing radical formation in cancer cells (e.g., disruption of heme degradation pathway, NO synthase inhibition or HSP90 modulation) have been described (reviewed in [9]). Thus, our first focus was to find miRs significantly correlating with PDT efficiency and sharing targets in these pathways, evaluating two hypotheses: (i) some overexpressed miRs in PDT insensitive cancer cells (“resistive-miRs”) may not only reflect a higher possibility of adapting and surviving the intrinsic ROS stress in these cells, they could furthermore play a direct role in evasion of PDT induced apoptosis and may serve as therapeutic targets via inhibition; *Vice versa*, (ii) overexpression of “phototox-miRs” could play an important role in achieving a better effect of PDT in these insensitive cell lines. Of note, our approach does not identify miRs *per se*, which are causally related with phototoxicity or PDT insensitivity. Therefore, to better define the role of “resistive-” and “phototox-miRs” in cell susceptibility towards ROS and hypoxia following PDT, we tried to identify and compare their mRNA targets following a stepwise bioinformatic approach.

*In silico* target prediction and computational methods providing statistics for the resulting lists of putative target genes using the Gene-Ontology (GO) database is established to manage large scale microarray results [43]. Numerous target prediction algorithms and bioinformatic tools are available (reviewed in [44,45]). We chose miRWalk [19] and DAVID, [46] mainly for practical reasons: MiRWalk allows a comparative analysis by eight prediction programs and DAVID allows a biological module-centric analysis providing a wide range of heterogeneous annotation content, such as GO terms, protein domains and pathways. By this means, miRs having no or only few (enriched) targets in known PDT related pathways and mechanisms (as reviewed in [9,47,48,49]) could be ruled out.

Finally, target analysis following combined literature search and bioinformatic approach revealed eight miRs showing reasonable involvement in cytoprotective mechanisms following PDT (Table 3, see supplementary data file 6 (Table S1) for a detailed list with annotations). “Phototox-miRs” 130a, 93* and 25 showed clear involvement in PDT insensitivity mechanisms corresponding to hypothesis (ii): overexpression of miR-130a could theoretically lead to more efficient PDT in resistive cell lines affecting ROS detoxification (GSH, Sestrins, [49,50], PRDX3, [51]), adaptation to ROS stress (destabilizing/decreasing HIF-1α [52,53,54,55], SIRT7 and SIRT6, [47,56]), down-regulation of pro-angiogenic factors (enriched targets in the GO term “positive regulation of angiogenesis”, [55,57], NOS3, [58,59,60,61]) and survival signaling, such as Akt/PI3-K and NF-κB activation [62,63,64,65]. Furthermore, no contradictive results, *i.e.*, targets, which would possibly reduce PDT efficiency if inhibited by this “phototox-miR”-130a, could be found. Similarly, “phototox-miR”-25, which showed the highest level of significance in correlation with PDT toxicity, could theoretically affect ROS detoxification targeting, HMOX2 and SESN3 [49,50], apoptotic signaling (BCL2, [66,67,68], NOX4, [69,70,71] and survival signaling following oxidative stress (Akt/PI3-K and NF-κB activation). Induction of G1/S arrest via Wnt-inhibition on the level of β-catenin by miR-25 [25] could theoretically intensify phototoxicity following PDT (shown for rapamycin *in vitro* [72]). Mir-93* showed repression of oxidative defence genes (*Nrf2* and *Sp1* [28,29]) and PTEN/Akt signaling pathway [30].

Summarizing these results, “phototox-miRs”-130a, 93* and 25 could contribute to a pro-apoptotic function in the context of PDT, if overexpressed, thus being a therapeutic candidate target for achieving a better effect in PDT-resistive BTC cell lines. Interestingly, phototox-miR-130a was the only miR in our study correlating significantly with GSH levels (negative correlation). GSH levels in the BTC cell lines correlated negatively—however, not reaching statistical significance with the phototoxic efficiency of mTHPC in our previous studies [13].

“Resistive-miRs” 20a*, 141, 200a, 200c and 203 revealed targets involved in ROS detoxification (e.g., HMOX1 indirectly via BACH1, [9,73,74,75,76,77]) adaptation to ROS stress (e.g., NOX1, [78]), apoptotic signaling (e.g., BAX, [79,80,81]) and survival mechanisms (e.g., KEAP1, [82]). Their targets are enriched in GO terms “apoptotic mitochondrial changes”, “release of cytochrome c from mitochondria”, “positive regulation of MAPKKK cascade”, “regulation of JNK cascade” and “regulation of stress-activated protein kinase signaling pathway”. In summary, these five “resistive-miRs” could play a direct role in evasion of PDT induced apoptosis and serve as potential therapeutic targets, if inhibited (hypothesis (i)).

However, some conflicting results could be found for these “resistive-miRs”: targets of miRs 20a*, 200c and 203 were enriched in the GO term (positive) “regulation of nitric-oxide synthase activity” and “positive regulation of nitric oxide biosynthetic process”. As *cytoprotective* roles of NO and NOS were postulated in the context of PDT [83,84], these results could indicate a *pro-apoptotic* function of these “resistive-miRs”. Interestingly, also HIF-1α (miRs 141 and 20a*), VEGFA, 70 kDa HSPA4L [9,85,86,87] and LOX [88] (all targets of miRs 141 and 200a) could implicate a theoretically controversial effect in the context of PDT if down-regulated by these miRs.

#### 2.2.2. Cancer Phenotype and Susceptibility to PDT

Phototoxicity of PDT in our set of human biliary tract cancer cell lines seemed to be highly heterogeneous and preferentially linked to low differentiation and high proliferation of the cells [12]. PDT was particularly effective in cells with high levels of eight “phototox-miRs” and (except for miRs 25, 130a, 489 and 455) a phenotype characterized by high expression of the mesenchymal marker vimentin and high proliferation. A PDT-resistive phenotype was characterized by high expression of miR-200 family members, miR-203 and (in case of miR-cluster 200a/b-429) expression of differentiation markers Ck19 and Ck8/18. Enforced expression of the miR-200 family members repressed epithelial-mesenchymal-transition (EMT) by targeting and down-regulating ZEB1 and ZEB2 directly, resulting in enhanced E-cadherin expression and inhibition of tumor cell migration and cancer cell motility *in vitro* and *in vivo* as demonstrated previously [89,90] and reviewed in [34]. Summarizing these studies, a negative impact on EMT and a close association with a less malignant phenotype, which is less vulnerable to oxidative stress, could explain the high expression of miR-200 family members associated with PDT insensitivity in our set of BTC cell lines. Impaired redox balance and consecutive intrinsic oxidative stress in cancer are thought to play an important role not only in cell proliferation and genetic instability, but also in survival strategies: cells that are equipped with a flexible protective machinery are maintaining redox homeostasis in a highly dynamic state and have a higher possibility of adapting and surviving this oncogenic ROS stress ([49,91], reviewed in [47]). Examples are adaptation to high ROS levels in aggressive cancer phenotypes via regulation of intracellular ROS by CD44 [38,92], and evasion of anoikis, leading to EMT in a ROS-mediated manner ([93,94,95,96], reviewed in [97]). CD44, which is a validated target of miR-200c [36,37], might play a crucial role in the regulation of intracellular ROS, thus contributing to adaption of cancer stem cells (CSC) undergoing EMT to a relatively high level of intracellular ROS and leading to metastasis and drug resistance. Of note, CD44, together with CD133 could be recently shown to have an impact on prognosis in BTC [98]. In a study with nasopharyngeal carcinoma CSCs, the CD44 antigen was associated with an aggressive phenotype, EMT and elevated intracellular ROS levels [38]. MiR-200c suppressed *in vivo* tumor growth accompanied by elevated E-cadherin levels and down-regulated CD44 expression [36]. The down-regulation of miR-203 by CD44 signaling seemed to be critical for stemness-maintenance in human colon CSCs [40]. These publications underline the ROS-protective function of CD44 in malignant cancer phenotypes and may explain the suppression of “resistive-miRs” 200c and 203 in PDT-susceptible BTC cell lines.

In summary, target prediction and bioinformatic approach limited the total number of 20 miRs correlating significantly with PDT effectiveness to eight candidate miRs showing involvement in underlying mechanisms of PDT insensitivity, oncogenic ROS stress and differences in cancer phenotype. Future research based on the experiments described here should include validation of *in silico* predicted targets by comprehensive mRNA profiling via array-based expression platforms and verification of these targets by protein expression analysis. Additionally, functional analyses are required to prove a causative association of the expression of particular miRs and the cellular susceptibility towards PDT by inhibition or over-expression of such candidate miRs.

## 3. Experimental Section

### 3.1. Substances and Cell Culture

An *in vitro* model of *n* = 8 BTC cell lines was used as described in [12,13,24]. Bile duct carcinoma cell lines CCSW-1 (G2), BDC (G4), Egi-1 (G3), SkChA-1 (G3), TFK-1 (G2) and gallbladder cancer cell lines MzChA-1 (G1), MzChA-2 (G2), GBC (Wittier, G1) (see [99] for references) were cultured as described previously [12,13,24] using Dulbecco’s modified Eagle’s medium (DMEM) supplemented with 10% (*v*/*v*) fetal bovine serum (FBS; PAA Laboratories, Pasching, Austria) and are collectively referred to as biliary tract cancer (BTC) [18] cell lines. For photosensitizer (PS) incubation and post-illumination periods, serum-free DMEM (sf-DMEM) with otherwise identical supplements was used. For all experiments cells were seeded at cell densities of 3.68 × 10^4^ (for BDC, MzChA-2), 4.41 × 10^4^ (for CCSW-1, GBC), 5.15 × 10^4^ (for SkChA-1), 5.88 × 10^4^ (for Egi-1, TFK-1), and 6.62 × 10^4^ (for MzChA-1) per cm^2^ using 4 mL and 100 µL 10% FBS DMEM for 6 cm petri dishes and 96-well microplates, respectively.

### 3.2. Photodynamic Therapy and Phototoxicity Experiments

MTHPC was obtained from Biolitec AG (Jena, Germany) at a concentration of 5.88 mM (4 mg·mL^−1^ diluted with the ethanol/propylene glycol solvent to a working stock concentration of 0.588 mM (400 µg·mL^−1^) and stored at 4 °C in the dark. Porfimer sodium was obtained from Seehof Laboratorium (Wesselburenerkoog, Germany) at a concentration of 9.96 mg·mL^−1^ (corresponding to 16.67 mM based on a nominal molecular weight of 600 g·mol^−1^ [99,100,101,102]) and stored at 4 °C in the dark. Resazurin sodium salt was obtained from Sigma-Aldrich (Vienna, Austria). Cells cultured in 96 well microplates (black walls, clear bottom) were washed once with 100 µL sf-DMEM and incubated with 0.588 µM (400 ng·mL^−1^) mTHPC, 1.2 µg·mL^−1^ (2 µM) porfimer sodium, respectively, again using 100 µL sf-DMEM [99]. Subsequent to 20 h incubation, photosensitized cells were illuminated from below the microplates using LED arrays as light sources. 660 nm (0.65 J/cm^2^) and 624 nm (4.30 J/cm^2^) dominant wavelength with a current of 5A were employed for 3 or 7 min (for mTHPC and porfimer sodium, respectively, described in [21]). Directly after illumination and 24 h afterwards, the cellular viability was assessed by adding 20 µL 2.5 mM resazurin [20] in PBS to each well, incubating for 2 h at 37 °C and measured using the Infinite M200 microplate reader (Tecan, Grödig, Austria) at λ_ex_ = 535 nm and λ_em_ = 588 nm [12,13]. Similar to other viability assays (MTT), the fluorimetric resazurin method indicates the proportion of metabolically active (viable) cells [20].

For the current data set (mTHPC and porfimer), the obtained values were corrected for blank wells and related to the baseline viability (0 h values); the second data set (mTHPC, adopted from an earlier publication [13]) was related to untreated controls measured at 24 h post illumination. For correlation of miR expression data with the phototoxicity after correction by the cell-dependent uptake of the PS, the fluorimetry uptake values were adopted from the mentioned earlier study [13]. For the current set of mTHPC- and porfimer sodium-based phototoxicity, the cellular uptake (corrected by total protein) was measured by fluorimertry as described previously [13]—see supplementary file 7 for raw data and corresponding diagrams.

### 3.3. MicroRNA Expression Profiling

MicroRNA expression profiling was carried out for every cell line in one replicate analysis (for cell lines MzChA-1, MzChA-2, SkChA-1 and TFK-1 in two independent replicates) using Taqman Array MicroRNA Cards (Human A + B Card Set v3; Applied Biosystems, Vienna, Austria) comprising 754 unique assays specific to human miRs preceded by RNA sample preparation using Megaplex RT Primers human pool set (v3.0) and Taqman Reverse Transcription System consistent with Sanger miRBase v14 (Applied Biosystems). Analyses were performed using the ViiA7 real-time qPCR instrument (Applied Biosystems). Small-nucleolar RNAs U6snRNA and RNU48 were chosen as endogenous controls. *C*_t_ values of a third potential control RNU44 showed considerable variation in cell lines TFK-1 and EGI-1 and was not expressed in cell line SkChA-1. *C*_t_ values >40 were discarded. Average *C*_t_-values of controls were subtracted from miR-*C*_t_-values to obtain (absolute) Δ*C*_t_-values. In case of two independent measurements, averaged 2^−Δ*C*t^-values were used (if *C*_t_-values of miRs differed by more than 2 cycles in two independent measurements, they were omitted). DataAssist™ v3.0 Software (Applied Biosystems) was used for visualization (heat map in Figure 3, global view and average linkage settings) and hierarchical clustering of miR-expression profiles based on the values using Pearson’s Correlation. Assignment to family classification and clustering were performed according to the Sanger miRBase v18.0. This set of data was evaluated by statistical analysis (as described below) to identify miR patterns that are linked with preferential high or low cellular sensitivity towards PDT.

### 3.4. Bioinformatical Approach and Literature Search

To identify miR targets we used the miRWalk database [19]. This database provides the possible miR binding sites on the complete sequence (promoter, 5' UTR, CDS (Coding DNA Sequence) and 3' UTR) of known genes and three complete mitochondrial genomes. For target prediction we set the following preferences as default: 2000 bp upstream flanking, 5'-, 3'-UTR and CDS, seven nucleotides minimum seed length, *p*-value 0.01 for distribution probability of random matches of a subsequence in the given sequence. MiRWalk was also used to search for validated targets. In case of miR-516b-3p no targets could be found in miRWalk, so alternatively miRDB prediction database (version 4.0) was used [23,103].

In order to identify Gene-Ontology (GO) categories in which miR targets are over-represented, we used the DAVID bioinformatics microarray analysis tool “functional annotation chart” [46]. In brief, this tool provides, among other functions, gene-GO term enrichment analysis to highlight the most relevant GO terms associated with a given gene list [45]. Fold Enrichment values were calculated defined as the ratio of the two proportions (input targets and background information). Additionally, for each GO term an EASE score (a modified Fisher exact *p*-value) was calculated representing the probability that the observed numbers of hits could have been obtained randomly. To achieve high stringency in the identification of miR targets we set the cut off *p*-value at 0.01. To make *p*-values better comparable among target-sets of different miRs (*p*-values are influenced by the absolute number of targets in a set), we calculated a score based on the *p*-value ranking percentage, “*p*-rank%”, defined as the tenfold of the reciprocal of *p*-value ranking percentage [10/(ranking of p-value of GO term × (100/n predicted and validates targets))], *i.e.*, a *p*-value ranking percentage of 50% corresponds to *p*-rank% = 0.2, and a *p*-value ranking percentage of 10% corresponds to *p*-rank% = 1. Contradictive or less specific results (e.g., GO terms as “response to UVA”, “regulation of MAP kinase activity” or TGF-β related terms and targets) were not taken into account.

Literature search was performed via PubMed: articles obtained by using the search terms “redox”, “cancer”, “reactive oxygen species” and “survival” (750 hits), “microRNA” and “redox” (195 hits) and “photodynamic therapy” and “cytoprotective” (21 hits) were screened for experimentally validated miR targets involved in cytoprotective mechanisms following oxidative stress (particularly PDT) in cancer. Without claim to completeness, the validated miR targets identified by this search strategy were presented in Table 3 and supplementary file 6 (Table S1) and discussed in the discussion section.

### 3.5. Statistics

Cytotoxicity analyses (cell line-dependent characterization of phototoxicity) included mean values of at least three independent experiments ± SEM. Multiple values within each experiment were corrected for outliers (Grubb’s test). MiR expression profiling included one to two biological replicates employing the Array Card system. Correlation analysis was performed (i) by comparison of raw viability data, PS uptake (measured in a previous study [13]) and uptake-corrected phototoxicity with miR expression patterns (2^−Δ*C*t^-values) according to Pearson product-moment correlation coefficient; and (ii) by comparison of miR expression patterns (2^−Δ*C*t^-values) with cellular characteristics (proliferation and differentiation markers) which were measured in a previous study [24] according to Spearman, respectively, using PASW Statistics 17.0 (SPSS GmbH Software, Munich, Germany). For all calculations, *p* < 0.05 and *p* < 0.01 was considered as significant or highly significant, respectively.

## 4. Conclusions

This project comprised an investigation on the role of the miRNome in determining the efficiency of PDT among eight biliary tract cancer (BTC) cell lines. Subsequent *in silico* target prediction and bioinformatical approach revealed eight miRs potentially involved in regulation of the cellular mechanisms of PDT. Therefore, overexpression of “phototox-miRs” 130a, 93* and 25 or inhibition of “resistive-miRs” 20a*, 141, 200a, 200c and 203 could improve susceptibility towards PDT in otherwise relatively insensitive cell lines. Based on functional verification in subsequent studies, this may be a useful strategy to increase the phototoxicity of PDT in cases following profiling of the most relevant miRs and rational patient stratification.

## Supplementary Materials

Supplementary materials can be found at http://www.mdpi.com/1422-0067/15/11/20134/s1.
